# An HSV-2 Trivalent Vaccine Is Immunogenic in Rhesus Macaques and Highly Efficacious in Guinea Pigs

**DOI:** 10.1371/journal.ppat.1006141

**Published:** 2017-01-19

**Authors:** Sita Awasthi, Lauren M. Hook, Carolyn E. Shaw, Bapi Pahar, Jacob A. Stagray, David Liu, Ronald S. Veazey, Harvey M. Friedman

**Affiliations:** 1 Infectious Disease Division, Department of Medicine, Perelman School of Medicine, University of Pennsylvania, Philadelphia, Pennsylvania, United States of America; 2 Division of Comparative Pathology, Tulane National Primate Research Center, Covington, Louisiana, United States of America; Louisiana State University Health Sciences Center, UNITED STATES

## Abstract

A genital herpes vaccine is urgently needed to prevent pain and suffering, reduce the incidence of neonatal herpes, and decrease the risk of HIV acquisition and transmission that accompanies genital infection. We evaluated a trivalent HSV-2 subunit antigen vaccine administered with CpG and alum in rhesus macaques and guinea pigs. The vaccine contains glycoproteins C, D and E (gC2, gD2, gE2) to block virus entry by gD2 and immune evasion by gC2 and gE2. In rhesus macaques, the trivalent vaccine induced plasma and mucosa neutralizing antibodies, antibodies that block gC2 and gE2 immune evasion activities, and stimulated CD4 T cell responses. After intravaginal challenge, a self-limited vaginal infection of brief duration was detected by histopathology and immunohistochemistry in naïve, but not in trivalent immunized macaques. Vaccine efficacy was evaluated in female guinea pigs. Animals were mock immunized, or immunized with gD2, the trivalent vaccine or the trivalent vaccine followed by a booster dose of gD2 (trivalent + gD2). The trivalent and trivalent + gD2 groups were 97% and 99% efficacious, respectively in preventing genital lesions and both outperformed gD2 alone. As a marker of transmission risk, vaginal swabs were evaluated daily for HSV-2 DNA and replication competent virus between five and seven weeks after challenge. HSV-2 DNA shedding was reduced in all groups compared with mock. Shedding of replication competent virus occurred on fewer days in the trivalent than gD2 immunized animals while the trivalent + gD2 group had no shedding of replication competent virus. Overall, the trivalent group had genital lesions on < 1% days and shedding of replication competent virus on 0.2% days. The vaccine has outstanding potential for prevention of genital herpes in humans.

## Author Summary

Approximately a half-billion people worldwide are infected with herpes simplex virus type 2 (HSV-2), the virus that causes genital herpes. In some individuals, infection results in painful, recurrent genital ulcers, while in others, the infection remains quiescent. In both settings, infected individuals may transmit virus to their intimate partners. Genital herpes increases the risk that an infected person will acquire HIV if exposed during sexual intercourse. A vaccine for the prevention of genital herpes is a high priority. We describe a vaccine that induces antibodies that block the ability of the virus to enter cells and that prevents the virus from escaping immune attack mediated by antibody and complement. The vaccine contains HSV-2 glycoproteins C, D and E and is immunogenic in non-human primates. The vaccine protects immunized non-human primates against a mild vaginal infection that develops in naïve animals after intravaginal inoculation of virus. Naïve guinea pigs develop severe genital disease, while immunized animals are almost 100% protected after intravaginal infection. The vaccine greatly reduces the number of days during the recurrent phase of infection that animals shed virus in genital secretions, thereby reducing the risk of transmission. We consider this novel vaccine a leading candidate for clinical trials aimed at preventing genital herpes infection in humans.

## Introduction

A half-billion people are infected with genital herpes worldwide [1]. The prevalence of genital herpes is 17% in the U.S. population between the ages of 15 and 49 years, and approximately 2-fold higher in sub-Saharan African countries [2]. Genital herpes increases transmission and acquisition of HIV-1 infection by 3- to 4-fold, and poses a risk of 3 per 100,000 live births that infants will develop neonatal herpes during delivery with a mortality of 19% [3–7]. Genital herpes infection is emotionally upsetting for many individuals based on concerns of transmission to their partners [8–10]. A vaccine for genital herpes is urgently needed, yet none is available. Researchers are pursuing a herpes vaccine with the goal of preventing genital lesions and subclinical infection, which is typically measured by genital shedding of HSV-2 DNA [11]. The importance of subclinical infection is that it accounts for much of the sexual transmission of genital herpes [8, 12].

Chiron Corp. evaluated a prophylactic vaccine containing two HSV-2 glycoproteins involved in virus entry, glycoproteins B (gB2) and D (gD2) given with MF59 as adjuvant [13]. The vaccine did not protect seronegative partners from HSV-2 infection, although it delayed onset of infection over the first 5 months after immunization. GlaxoSmithKline (GSK) assessed a prophylactic vaccine using gD2 antigen with monophosphoryl lipid A (MPL) and alum as adjuvants [14]. Overall, no protection against genital lesions was detected, although significant protection was noted in a subgroup of HSV-1 and HSV-2 doubly seronegative women. A follow-up trial was performed in doubly seronegative women that showed no overall protection against genital herpes; however, the vaccine was efficacious against HSV-1. This result was noteworthy because HSV-1 accounted for 60% of genital herpes infections in the control group [15]. Our interpretation of these studies is that targeting a vaccine to block HSV-2 entry is not sufficient. Our approach is to supplement blocking entry with a strategy to prevent immune evasion from antibody and complement.

HSV-1 and HSV-2 gC are immune evasion molecules that function as regulators of the complement cascade [16–19]. During complement activation, C3, the most abundant complement protein, is cleaved to C3b, which activates the membrane attack complex leading to virus neutralization and lysis of infected cells [20]. C3b stimulates B- and T-cell responses and serves as a link between innate and acquired immunity [21, 22]. HSV-1 and HSV-2 gC bind C3b to inhibit activities mediated by C3b [18, 23]. Immunization with gC1 and gC2 produces antibodies that bind to the glycoprotein and block its immune evasion functions [16, 24].

HSV-1 and HSV-2 glycoprotein E (gE) function as immune evasion molecules by binding the Fc domain of an IgG molecule that is bound by its F(ab’)_2_ domain to its target [10, 25]. A vaccine containing gE2 subunit antigen produces antibodies that bind to gE2 and block its immune evasion functions [10]. HSV-2 gC2 and gE2 perform activities similar to mammalian complement and IgG Fc regulatory proteins, yet share no sequence homology with mammalian receptors, which suggests virtually no risk that immunization will induce autoimmunity [26]. Here we demonstrate that a vaccine containing gC2, gD2 and gE2 is immunogenic in rhesus macaques and provides protection against genital lesions, shedding of HSV-2 DNA and shedding of replication competent virus in guinea pigs.

## Results

### Rhesus Macaque Antibody Responses

**Fig 1:**
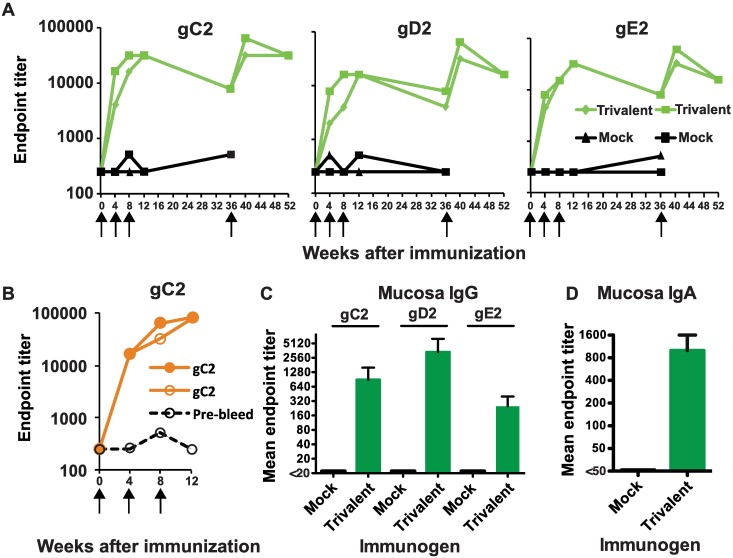
Rhesus ELISA antibody responses. **(A)** Plasma antibody endpoint titers induced by the trivalent vaccine. **(B)** ELISA antibody titers in animals immunized with gC2 alone. Each animal in (A) and (B) is shown separately. Arrows indicate immunizations. **(C)** Vaginal mucosa IgG gC2, gD2 and gE2 ELISA titers to the trivalent vaccine. **(D)** Vaginal mucosa IgA responses to the trivalent vaccine were tested against the three antigens, gC2, gD2 and gE2 added to a single well. Mock vaccine responses in (C) and (D) were evaluated after the third immunization at week 12, while trivalent vaccine responses were assessed after the booster dose at week 40. Results in (C) and (D) are the mean and SEM of two animals in each group.

We evaluated the immunogenicity of HSV-2 gC2/gD2/gE2 administered with CpG and alum (trivalent vaccine) in rhesus macaques. Two animals received CpG and alum (mock group) and two received the trivalent vaccine. Immunizations were given three times at monthly intervals. The rhesus macaques immunized with the trivalent vaccine produced robust IgG responses to each antigen that waned slightly over a six-month period (Fig 1A). At week 36, the trivalent group received a booster dose of the trivalent vaccine while the two mock-immunized animals were repurposed to receive three monthly injections of gC2 with CpG/alum. The titers rose briskly by week 40 to a higher peak level after the booster dose of the trivalent vaccine (Fig 1A), while animals immunized with gC2 alone produced high titers of gC2 antibodies by week 12 (Fig 1B).

The rhesus macaques immunized with the trivalent vaccine were evaluated for vaginal mucosal IgG and IgA ELISA antibody responses. Animals developed IgG responses to all three immunogens with the most robust response to gD2 (Fig 1C). The mucosa IgA response was tested against all three immunogens in a single well based on insufficient material to test each antigen individually. Both trivalent-immunized animals mounted IgA responses (Fig 1D).

**Fig 2:**
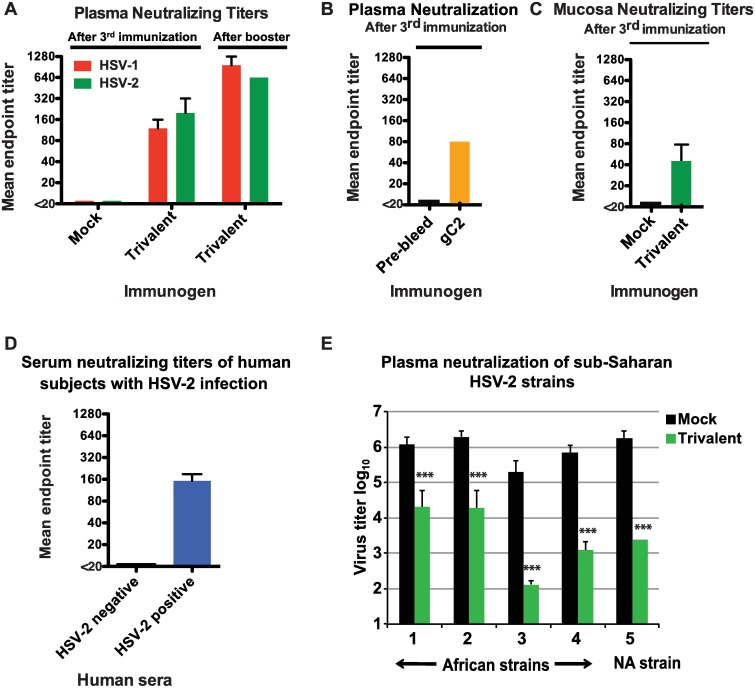
Rhesus neutralizing antibody responses. **(A)** Plasma neutralizing titers induced by the trivalent vaccine against HSV-1 and HSV-2 after the third immunization at week 12 and after a booster dose at week 40. **(B)** Neutralizing antibody titers induced by gC2 alone after the third immunization. Results are the mean of two animals. Pre-bleed represents serum obtained prior to the first gC2 immunization. **(C)** Mucosa neutralizing antibody titers to HSV-2 after the third immunization. Results are the mean and SEM of two animals. **(D)** Serum neutralizing antibody titer to HSV-2 in 10 human HSV-2 seropositive subjects with chronic HSV-2 infection and three HSV-2 seronegative controls. **(E)** Neutralization of sub-Saharan HSV-2 strains and a North American strain. 10^5^ to 10^6^ PFU of four HSV-2 strains from Uganda, Africa and one from North America (NA) were incubated with plasma from one mock (after the third immunization at week 12) or one trivalent-immunized animal (after the booster dose at week 40). Results represent the mean + SEM of three separate determinations. P values were calculated using mixed effects models to compare the mean responses. Repeated tests within an animal were adjusted for using a nested correlation structure. Sidak p-values were calculated to adjust for the multiple comparisons. *** indicates P<0.001.

We assessed the plasma neutralizing antibody responses against HSV-1 and HSV-2. HSV-1 is approximately as common as HSV-2 as the cause of first time genital herpes infection [27]. Plasma neutralizing antibody titers against both HSV-1 and HSV-2 were detected in the trivalent immunized animals after three immunizations that increased after a booster dose (Fig 2A). We evaluated whether animals immunized with gC2 alone produced plasma neutralizing antibodies against HSV-2. Neutralizing antibodies were detected (Fig 2B), although titers were lower than in trivalent immunized animals (Fig 2A). Animals immunized with the trivalent vaccine produced vaginal mucosa neutralizing antibodies (Fig 2C). For comparison purposes, we evaluated neutralizing antibody titers in sera of 10 human subjects with chronic HSV-2 infection (HSV-2 seropositive, HSV-1 seronegative) and 3 controls (HSV-1 and HSV-2 seronegative). The HSV-2 mean endpoint neutralizing antibody titer was 1:160 (Fig 2D), which was comparable to the titers achieved in rhesus macaques after the third immunization with the trivalent vaccine, yet 4-fold lower than titers after the booster dose (Fig 2A).

Genital herpes increases susceptibility to HIV infection [3]. The HIV epidemic is most intense in sub-Saharan Africa; therefore, we evaluated the breadth of the neutralizing antibody response induced by the trivalent vaccine against four HSV-2 isolates from sub-Saharan Africa (Uganda), and one isolate from North America, which is the same North American strain that was used in Fig 2A–2D. Neutralization was measured by the log_10_ titer reduction assay since we had insufficient plasma available to measure mean endpoint titers. In our experience the two assays yield comparable results [28]. Plasma from the trivalent-immunized animal neutralized the four HSV-2 sub-Saharan African isolates by 1.8–3.2 log_10_, while the North American strain was neutralized 2.8 log_10_ (Fig 2E). Therefore, the trivalent vaccine induced neutralizing antibodies to multiple African isolates.

**Fig 3:**
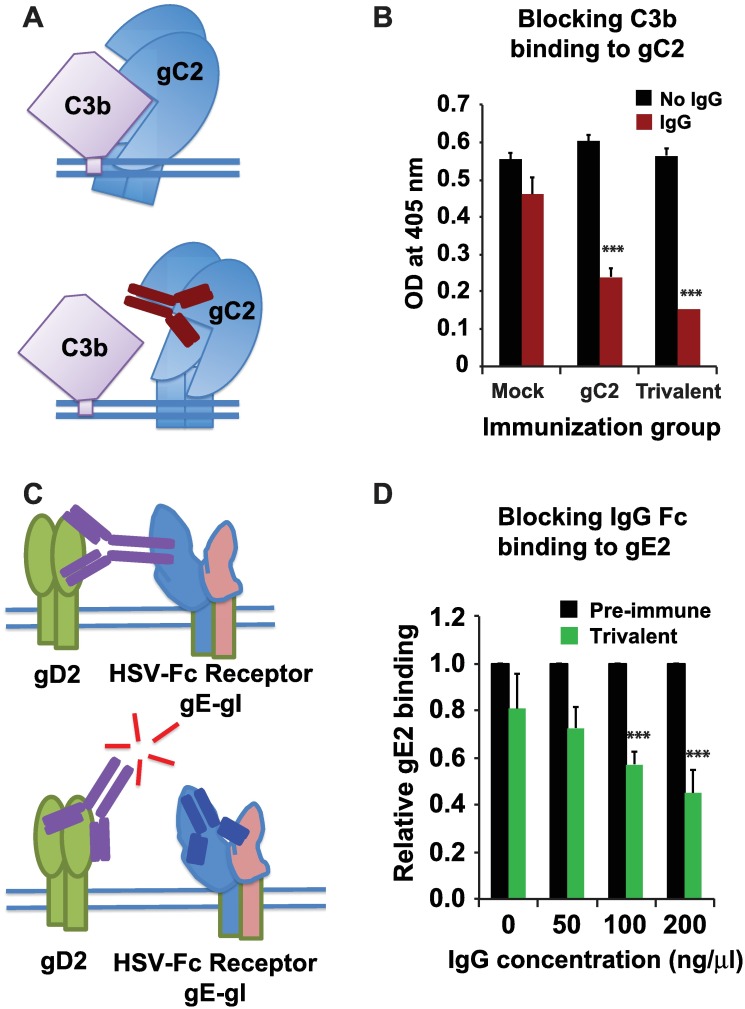
Antibodies that block immune evasion activities. **(A)** Top model: gC2 is a complement regulatory molecule. Activation of complement leads to deposition of complement component C3b on the virus or infected cell surface where gC2 binds to C3b and blocks downstream complement activation [31]. Bottom model: Antibody produced by immunization with gC2 binds and blocks its interaction with C3b, enabling downstream activation of complement [16]. **(B)** Blocking immune evasion domains on gC2. IgG obtained at week 12 after the third immunization of animals in the mock, gC2, or trivalent group was incubated with gC2 protein and added to C3b-coated wells. Results represent the mean + SEM of 2 IgG samples per group run 3 times. **(C)** Top model: gE2 is an IgG Fc binding molecule: An antibody produced by immunization with gD2 binds by its F(ab’)_2_ domain to gD2 and by its Fc domain to gE2 that forms an IgG Fc receptor complex with glycoprotein I (gI2) [25]. Bottom model: antibody to gE2 produced by immunization binds and blocks its ability to interact with the Fc domain of antibody to gD2, enabling the Fc domain to activate complement and mediate antibody dependent cellular cytotoxicity [29, 32]. **(D)** Blocking immune evasion domains on gE2. IgG obtained prior to the first immunization or at week 40 after the fourth immunization in the trivalent group was incubated with gE2 and then added to wells coated with nonimmune human IgG. Results are expressed relative to the binding of gE2 when incubated with IgG obtained prior to the first immunization. The results represent the mean + SEM of one pre-immune IgG and two immune IgG samples tested three times. P values for (B) and (D) were calculated using mixed effects models as described in the legend to Fig 2E. *** indicates P<0.001.

We evaluated whether immunization with the trivalent vaccine induces antibodies to gC2 and gE2 that block their immune evasion activities (concepts depicted in models, Fig 3A and 3C). IgG was purified from plasma obtained from mock, gC2, and trivalent immunized animals, incubated with gC2 and added to C3b coated wells. We previously reported that blocking C3b binding to gC2 occurs in a dose-dependent fashion [29]. Here, we evaluated an IgG concentration of 125μg/100μl that is equivalent to a 1:160 dilution of rhesus macaque serum [30]. At 12 weeks, the IgG from mock-immunized animals failed to prevent gC2 binding to C3b while IgG from gC2 and trivalent immunized animals significantly blocked gC2 binding to C3b (P<0.001 comparing gC2 and trivalent with mock) (Fig 3B). We next evaluated whether antibodies induced by the trivalent vaccine block the interaction between gE2 and the IgG Fc domain. Blocking by rhesus nonimmune IgG obtained prior to the first immunization was set at a relative value of 1.0 to distinguish blocking mediated by immune IgG (binds to gE2 at a higher affinity by the F(ab’)_2_ than by the Fc domain) and nonimmune IgG (binds to gE2 only by the lower affinity Fc domain) [10, 25]. IgG obtained prior to immunization (pre-immune) and after the fourth immunization of the trivalent vaccine at week 40 was evaluated at concentrations of 0, 50, 100 and 200ng/μl. A concentration of 200ng/μl represents approximately a 1:100 dilution of rhesus macaque serum [30]. The nonimmune IgG or trivalent IgG was incubated with gE2 and added to nonimmune IgG coated on wells. Immune IgG from trivalent-immunized animals blocked gE2 binding to nonimmune IgG in a dose-dependent manner (P<0.001 comparing immune IgG at 100ng/μl or 200ng/μl with immune IgG at 0ng/μl) (Fig 3D).

### Rhesus Macaque CD4^+^ T Cell Responses

**Fig 4:**
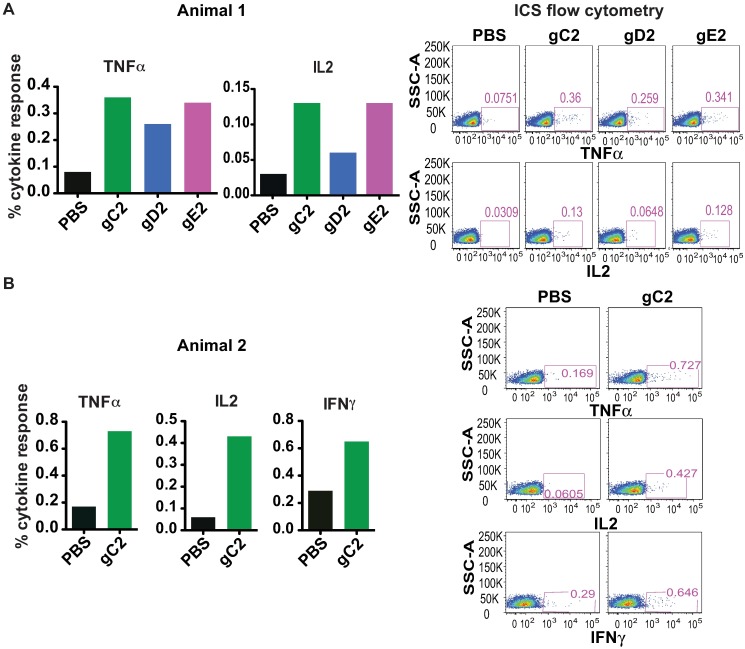
Rhesus CD4^+^ T cell responses to the trivalent vaccine. **(A-B)** PBMCs after the booster dose were stimulated *in vitro* with media, gC2, gD2 or gE2 subunit antigens. Results of each animal are shown separately. The intracellular cytokine staining (ICS) flow cytometry figures are shown to the right of each bar graph.

PBMCs were assayed for antigen-specific T cell responses prior to the first immunization (pre-immune) with the trivalent vaccine and after the booster dose at week 40. Neither animal demonstrated antigen-specific CD4^+^ T cell responses in pre-immune samples. Animals 1 and 2 were immunized with the trivalent vaccine. Animal 1 produced TNFα and IL2 responses when stimulated with gC2, gD2 and gE2 *in vitro* (Fig 4A). Animal 2 produced TNFα, IFNγ and IL2 responses when stimulated with gC2, but did not produce responses to gD2 or gE2 (Fig 4B). CD8^+^ T cell responses were not detected.

### Intravaginal Challenge of Naïve and Trivalent Immunized Rhesus Macaques

**Fig 5:**
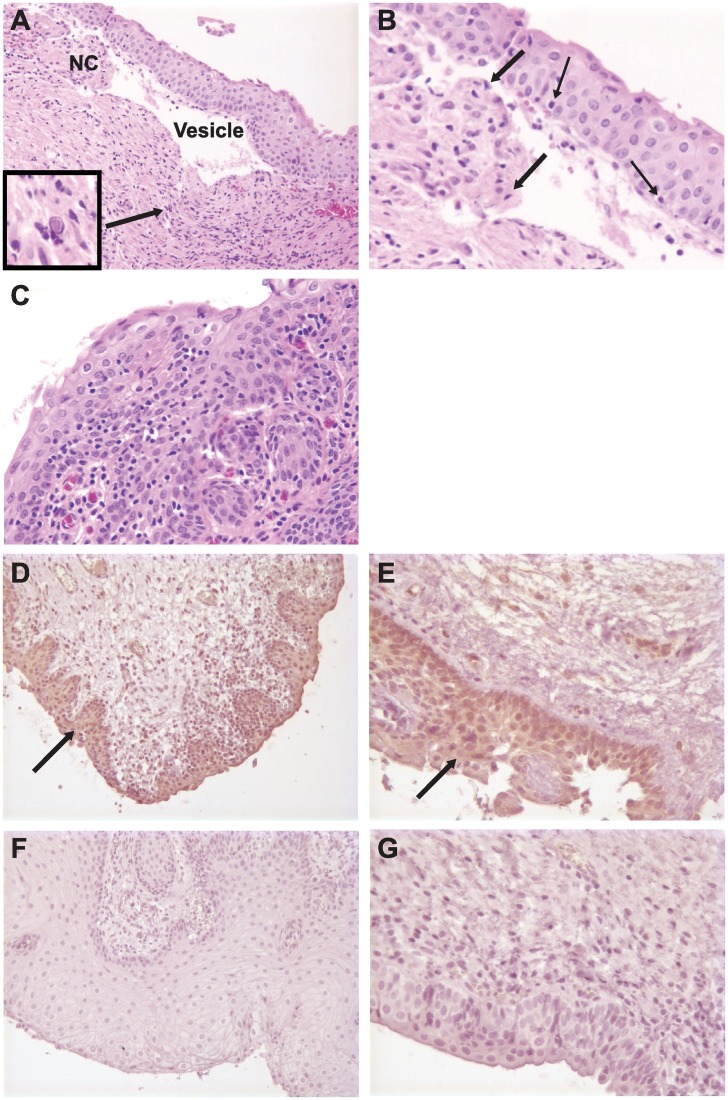
Histopathology and immunohistochemistry of vaginal mucosa in naïve and trivalent immunized rhesus macaques. **(A)** Histopathology of a naïve animal two weeks after intravaginal infection. An intraepithelial vesicle and necrotic cells (NC) were detected. Magnification 20X. The boxed area shows a magnified view of a typical HSV intranuclear inclusion that is located in the tissue at the tip of the arrow. **(B)** Computer-generated magnification of the necrotic cells in Fig 5A. Thin arrows indicate hypotonic, clear fluid-filled cytoplasm, while thick arrows identify pyknotic nuclear degeneration. **(C)** Histopathology of the second naïve animal demonstrating mucosa infiltration of many lymphocytes and plasma cells. Magnification 40X. **(D)** Immunohistochemistry demonstrating moderately strong HSV-2 antigen staining (arrow) of vaginal biopsy tissues from a naïve animal eight days after the third HSV-2 intravaginal challenge. Magnification 20X. **(E)** Moderately strong HSV-2 antigen staining (arrow) of another naïve animal taken at the same time as Fig 5D. Magnification 40X. **(F)** Immunohistochemistry demonstrating no HSV-2 antigen staining of vaginal tissues obtained from a trivalent immunized animal eight days after the third HSV-2 intravaginal challenge. Magnification 20X. **(G)** Negative staining for HSV-2 antigen in the second animal immunized with the trivalent vaccine taken at the same time as Fig 5F. Magnification 40X.

Two naïve macaques became available for intravaginal HSV-2 challenge. These animals and the two trivalent-immunized animals were challenged intravaginally with 1x10^8^ PFU of HSV-2 strain G four months after the trivalent group had received the booster immunization. No disease was noted by colposcopy of the vaginal vault. Vaginal biopsies were performed only on the two naïve animals 14 days after challenge and processed for histopathology. Unexpectedly, we detected vesicle formation, an HSV intranuclear inclusion, and areas of necrotic cells in one animal (Fig 5A and 5B), while mucosa infiltration of many lymphocytes and plasma cells were noted in the second animal (Fig 5C).

Two additional naïve animals became available eight months after the first challenge. These two animals were challenged for the first time and the prior four animals (two trivalent immunized and two naïve controls) were challenged for a second time using 2x10^9^ PFU HSV-2 strain G. None showed signs of illness. All six animals were challenged again two weeks later with the same dose of virus. Again, no animal became ill. Vaginal biopsies were performed eight days after this last challenge and processed for HSV-2 antigen staining by immunohistochemistry. The four naïve animals all demonstrated HSV-2 antigen in genital tissues (two representative naïve animals are shown in Fig 5D and 5E) while the two trivalent immunized animals were negative for HSV-2 antigens (Fig 5F and 5G). One of four naïve animals had low quantities of HSV-2 DNA detected by qPCR (1424 copies), while one of two immunized animals was positive for even lower quantities of HSV-2 DNA (636 copies). No residual HSV-2 antigen was detected by immunohistochemistry in the naïve animals when euthanized one week later.

### Guinea Pig ELISA and Neutralizing Antibody Titers

**Fig 6:**
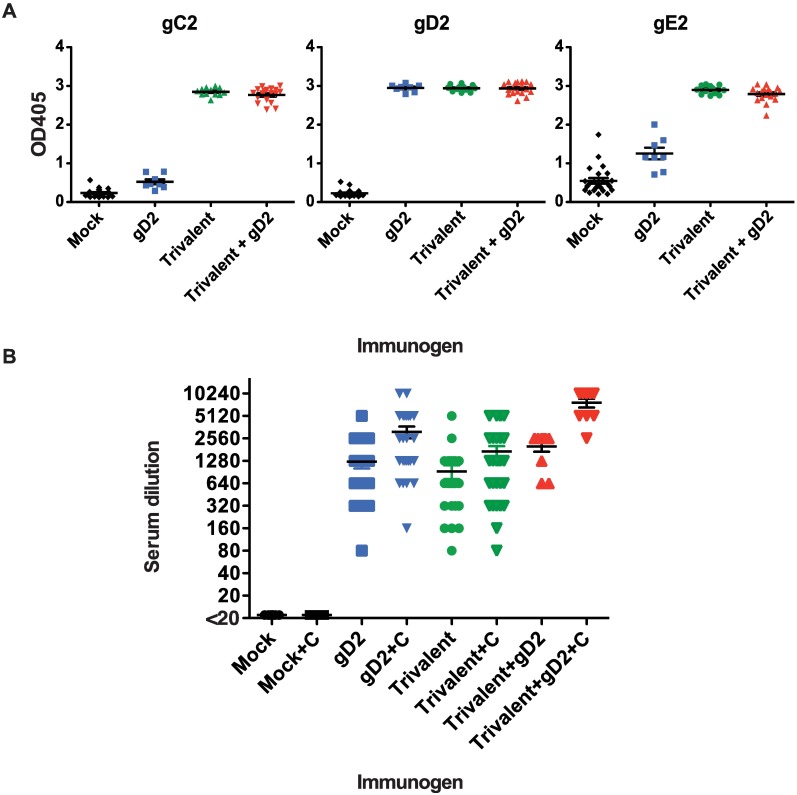
Guinea pig IgG ELISA and neutralizing antibody responses. **(A)** IgG ELISA titers were measured at a 1:1000 dilution of serum. Samples shown are from the second experiment. P values were calculated using Fisher’s exact test. **(B)** Serum neutralizing titers with and without complement. Samples evaluated were obtained two weeks after the final immunization from animals in the first and second experiments. P values within groups comparing with and without complement were calculated by Student’s t-test, while comparisons between groups were done using the Mann Whitney test.

Forty-one guinea pigs were immunized three times at two-week intervals with CpG/alum (mock group, n = 10), gD2 CpG/alum (gD2 group, n = 16), or gC2/gD2/gE2 CpG/alum (trivalent group, n = 15). In a second experiment 44 animals were immunized (mock, n = 14; gD2, n = 9; trivalent, n = 12) and a fourth group was added that received the trivalent vaccine followed two weeks later by a booster dose of gD2 CpG/alum (trivalent + gD2, n = 9). ELISA results for the second experiment are shown (Fig 6A) and are representative of both experiments. Animals immunized with gD2 alone had robust ELISA IgG responses to gD2, while animals immunized with the trivalent vaccine or the trivalent vaccine + gD2 had potent responses to all three antigens (P<0.001 comparing gC2, gD2 and gE2 titers in trivalent or trivalent + gD2 group with mock, or gD2 titers in gD2 group with mock, or gE2 titers in trivalent or trivalent + gD2 group with gD2 and mock; P values were not significant comparing gD2 titers in the trivalent, trivalent + gD2, or gD2 groups) (Fig 6A).

Neutralizing antibody titers were evaluated with and without complement and are shown for the two experiments combined. Neutralizing titers were significantly increased by complement in gD2, trivalent and trivalent + gD2 immunized animals (P = 0.0002 for gD2, P = 0.024 for trivalent, P = 0.0001 for trivalent + gD2) (Fig 6B). Neutralizing titers were higher in the gD2 alone group than in the trivalent group. Although not significant in the absence of complement (P = 0.32), the difference was significant in the presence of complement (P = 0.03). The diminished neutralizing antibody titers in the trivalent group suggested that the trivalent vaccine formulation may not be optimal at this time and led to our decision to include the trivalent + gD2 group in the second experiment. Neutralizing antibodies both with and without complement were higher in the trivalent + gD2 group than gD2 alone (without complement, P = 0.033 and with complement, P = 0.002), or the trivalent group (without complement, P = 0.003 and with complement, P<0.0001). The results indicate that complement enhanced the neutralizing titers of each group, and that the booster dose of gD2 administered to the trivalent group significantly increased the neutralizing titers of the trivalent group, both with and without complement.

### Guinea Pig Genital Lesions

**Fig 7:**
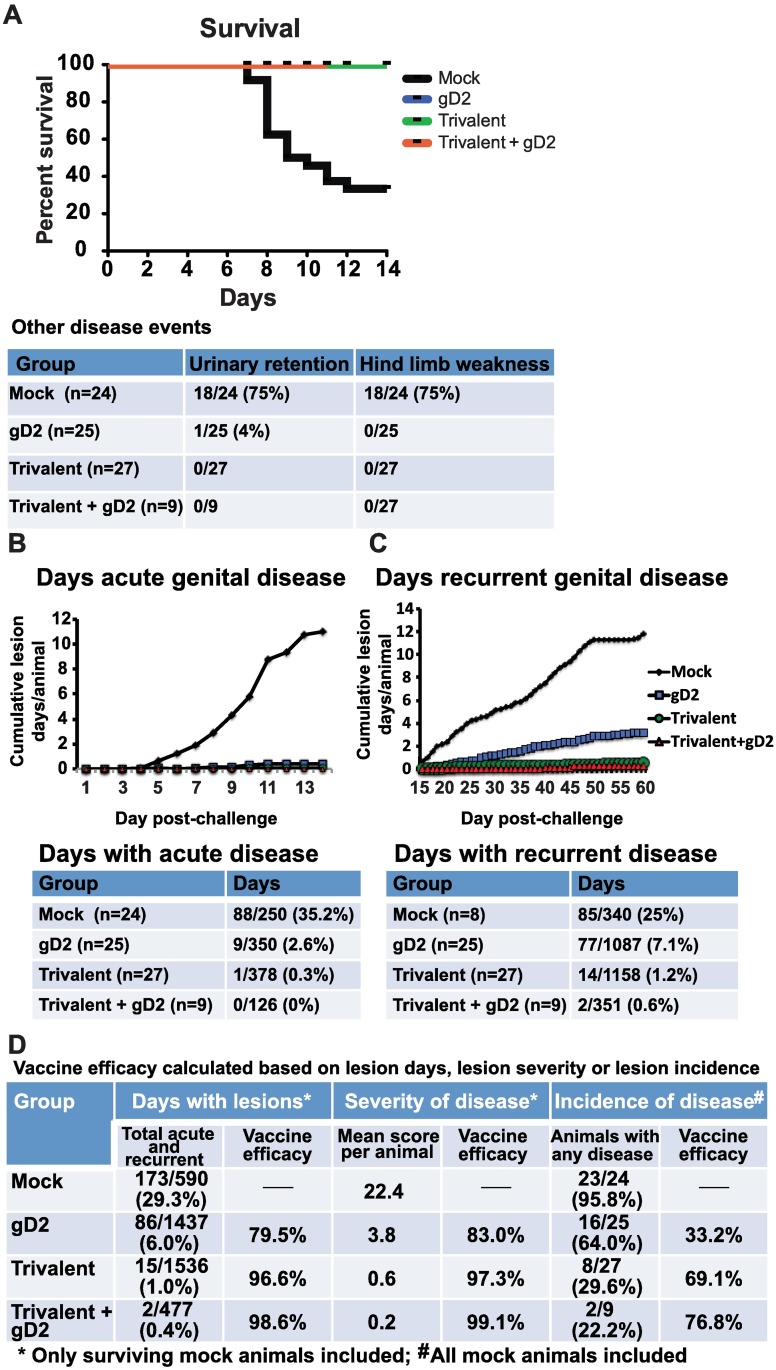
Survival and genital lesions in immunized guinea pigs after intravaginal challenge. **(A)** Survival curves. The table shows urinary retention and hind limb weakness during the first two weeks. **(B)** Days with acute genital lesions. **(C)** Days with recurrent genital lesions. Tables below (B) and (C) show the numbers used to generate the graphs. **(D)** Vaccine efficacy of gD2, trivalent, or trivalent + gD2 compared with mock. P values for (A) were calculated using Prism software by linear regression, while P values for (B-D) were calculated using Fisher’s exact test, except that severity of disease was calculated by Student’s t-test.

Vaccine efficacy was assessed using the female genital infection model [16]. Results shown are for the two experiments combined totaling 85 animals. Animals were challenged intravaginally with 5x10^5^ PFU of HSV-2 strain MS. All animals in the gD2, trivalent and trivalent + gD2 groups survived, while 8/24 (33%) in the mock group survived (P<0.001 compared with other groups) (Fig 7A). Eighteen of 24 (75%) animals in the mock group developed urinary retention and hind limb weakness, while 1 of 25 (4%) animals in the gD2 group developed urinary retention, and no animal in the trivalent (0/27) or trivalent + gD2 (0/9) group had these complications (P<0.001 comparing trivalent, trivalent + gD2, or gD2 group with mock) (Fig 7A table).

Our primary efficacy endpoint was the number of days animals had genital lesions. We scored guinea pigs for acute genital lesions on days 1–14 (Fig 7B), and recurrent genital lesions on days 15–60 (Fig 7C). The trivalent, trivalent + gD2, and gD2 groups each outperformed the mock group with fewer days of acute or recurrent genital disease (P<0.001). The tables associated with Fig 7B and 7C indicate that the trivalent and trivalent + gD2 groups also had fewer days of acute disease than the gD2 group (comparing trivalent with gD2, P<0.01; comparing trivalent + gD2 with gD2, P = 0.12, not significant) and fewer days of recurrent disease (comparing trivalent with gD2, P<0.001; comparing trivalent + gD2 with gD2, P<0.0001). Animals were scored for disease severity in addition to scoring for days with disease. The table in Fig 7D shows the vaccine efficacy for gD2, trivalent, and trivalent + gD2 groups compared with mock based on the number of days animals had genital lesions (both acute and recurrent), or based on lesion scores (severity of disease), or disease incidence (remaining disease-free) (Comparing days with lesions: gD2, trivalent or trivalent + gD2 with mock, P<0.0001; trivalent or trivalent +gD2 with gD2, P<0.0001. Comparing severity of disease: gD2 with mock, P = 0.01; trivalent with mock, P<0.01; trivalent + gD2 with mock, P = 0.07; trivalent with gD2, P<0.01; trivalent + gD2 with gD2, P<0.05. Comparing incidence of disease: gD2 with mock, P = 0.01; trivalent or trivalent + gD2 with mock, P<0.0001; gD2 with trivalent, P<0.05; gD2 with trivalent + gD2, P = 0.052). We conclude that the trivalent vaccine with or without the extra dose of gD2 is highly efficacious in preventing genital lesions.

**Fig 8:**
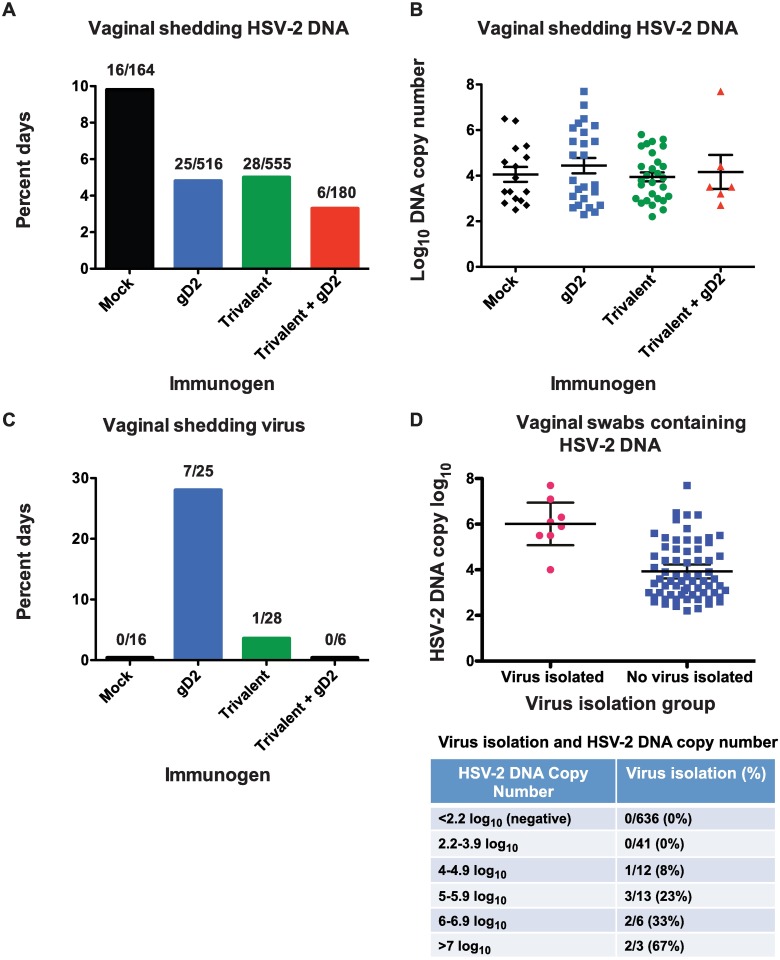
Vaginal shedding of HSV-2 DNA and replication competent virus in guinea pigs from days 29–49 post-challenge. **(A)** Percent of days with vaginal shedding of HSV-2 DNA. Results shown are from 8 animals in mock, 25 in gD2, 27 in trivalent, and 9 in trivalent + gD2 groups. P values were calculated using Fisher’s exact test. **(B)** Log_10_ HSV-2 DNA copy number during episodes of vaginal shedding of HSV-2 DNA. P values were calculated using the Mann Whitney test. **(C)** Percent days of vaginal shedding of virus. P values were calculated using Fisher’s exact test. **(D)** HSV-2 DNA copy number in vaginal swabs that yielded replication competent virus and vaginal swabs that failed to grow virus. P value was calculated by the Mann Whitney test. Table below (D): Isolation of replication competent virus at various HSV-2 DNA copy number in vaginal secretions. Only samples from the second vaccine experiment were cultured on days when no HSV-2 DNA was detected.

Guinea pig vaginal secretions were evaluated by qPCR from days 29–49 post-challenge for shedding of HSV-2 DNA and for shedding of replication competent virus. Only eight (33%) mock-immunized animals survived the acute infection; therefore, the surviving animals represent the least ill of the mock group. On days 29–49 post-challenge, HSV-2 DNA shedding was detected on 10% of the days in the surviving mock animals, 5% in the gD2 group, 5% in the trivalent group and 3% in the trivalent + gD2 group (P = 0.04 mock versus trivalent or gD2, P = 0.03 mock versus trivalent + gD2) (Fig 8A). No differences were detected comparing the HSV-2 DNA log_10_ copy number in any of the groups on days that animals were shedding HSV-2 DNA (Fig 8B). We performed virus cultures on the vaginal swab samples to further assess the possible infectivity of the genital secretions. Replication competent virus was cultured from 7/25 HSV-2 DNA positive samples in the gD2 group compared with 1/28 in the trivalent group (P = 0.02) and 0/6 in the trivalent + gD2 group (P not significant comparing trivalent + gD2 with mock or trivalent) (Fig 8C). In the mock group, 0/16 HSV-2 DNA positive samples yielded infectious virus, perhaps reflecting the milder infection in these animals (P = 0.03 mock versus gD2 group, P value not significant comparing mock with trivalent or trivalent + gD2 group) (Fig 8C). Cultures performed on days when vaginal swabs were negative for HSV-2 DNA were consistently negative for replication competent virus.

We evaluated the correlation of HSV-2 DNA copy number in vaginal secretions with isolation of virus from vaginal swab samples. Overall, on days that virus was recovered from vaginal secretions, the HSV-2 DNA copy number was 2.1 log_10_ higher than on days with no virus recovered (P<0.001) (Fig 8D). No animal shed virus when the HSV-2 DNA copy number was <4 log_10_. The probability of shedding replication competent virus increased with each log_10_ increase in HSV-2 DNA copy number (Fig 8D table).

As a marker of infection, we assayed dorsal root ganglia for HSV-2 DNA at the end of the experiment (≥ 60 days after infection). Among the eight mock immunized animals, only 3/8 had HSV-2 DNA detected, despite 7/8 developing genital lesions post-challenge. Among the 61 animals in the gD2, trivalent, or trivalent + gD2 immunization groups, only 2/61 had HSV-2 DNA, despite 24 animals developing genital lesions post-challenge. We generally detect a higher DNA copy number in ganglia and many more mock immunized animals have positive ganglia when samples are taken at earlier times post challenge, which suggests that HSV-2 DNA copy number in dorsal root ganglia may decline over time.

## Discussion

Our objectives for the rhesus studies were to assess the immunogenicity of the trivalent vaccine by determining whether immunization induced: i) mucosa gC2, gD2, and gE2 IgG and IgA ELISA and neutralizing antibodies; ii) high titers of plasma neutralizing antibodies; iii) antibodies that bind to gC2 and gE2 and block their immune evasion functions; and iv) CD4 T cell responses. All of these objectives were met. The trivalent vaccine also appeared to protect the animals against vaginal infection, although the results are more supportive than conclusive based on the small number of animals evaluated. Detecting evidence of HSV-2 vaginitis in naïve animals was unexpected since rhesus macaques are thought to be resistant to genital HSV-2 infection. We did not follow these animals long term by collecting vaginal swab samples to assess whether animals developed intermittent genital shedding of HSV-2 DNA, as previously reported [33]. Animals were treated with medroxyprogesterone to thin the vaginal mucosa prior to intravaginal challenge, and animals had vaginal biopsies after each challenge, which may have predisposed the animals to infection. Nevertheless, only the naïve animals developed histopathological and immunohistochemical evidence of HSV-2 infection, which suggests that the trivalent vaccine was protective. An effective HSV-2 vaccine has been estimated to reduce the risk of HIV infection in sub-Saharan Africa by 30–40% [34]. The rhesus genital infection model may prove useful to assess mechanisms by which HSV-2 increases the susceptibility to HIV [33].

Our goal for the guinea pig studies was to assess efficacy of the trivalent vaccine. We compared the trivalent vaccine with gD2 alone based on gD2 antigen being used in prior human trials that failed to achieve their primary endpoints [14, 15]. The gD2 subunit vaccine preparation used in our studies differs from the one administered in the human trials in that our gD2 antigen encompasses 306 amino acids after the signal sequence compared with the GSK strain that consists of 284 amino acids [35]. In addition, we used CpG/alum as adjuvants compared to MPL/alum used by GSK. MPL is a TLR4 agonist, while CpG is a TLR9 agonist [36, 37].

The gD2 antigen with CpG/alum was highly protective in guinea pigs; nevertheless, the trivalent vaccine significantly outperformed gD2 alone in preventing acute and recurrent genital lesions. Using lesion days as the primary endpoint, the vaccine efficacy for the trivalent immunogen was impressive (96.6% for trivalent and 98.6% for the trivalent + gD2 group). In contrast, using disease incidence, vaccine efficacy of the trivalent immunogen was less (69.1% for trivalent and 76.8% for trivalent + gD2 group). Using disease incidence as an indicator of efficacy fails to capture the impressive protection provided by the trivalent or trivalent + gD2 groups by giving equal weight to an animal that has a single lesion or to an animal with multiple lesions on many days. Uninfected female guinea pigs occasionally develop redness on their genitals that can be falsely scored as a genital lesion, which in our experience happens approximately 1 day in 200 (0.5%). These rare false positives can have a much greater impact on efficacy when calculating disease incidence than when considering disease days or disease severity.

The ELISA antibody titers increased after each immunization of macaques and reached their highest levels after a booster dose. Durable antibody titers persisted over 28 weeks between the third immunization and the booster dose. Whether these antibody titers are sufficient for long term protection against HSV-2 challenge is unknown as the immune correlates of protection against genital HSV-2 infection have not been defined [38]. Perhaps even higher titers and more durable responses would develop if different antigen and adjuvant concentrations were used or if the immunization schedule were similar to the one employed in human trials of 0, 1 and 6 months [14, 15] instead of the schedule of 0, 1 and 2 months used here.

In guinea pigs, we detected IgG binding to gE2 by ELISA using sera from mock-immunized and gD2-immunized animals. These results were expected as gE2 is an IgG Fc binding protein, including binding the Fc domain of non-immune IgG. Much higher ELISA gE2 IgG titers were noted in the trivalent and trivalent + gD2 groups, which indicates that the IgG F(ab’)_2_ domain bound to gE2 at higher affinity than IgG Fc domains. Regarding T cell responses, we did not detect CD8^+^ T cell responses in rhesus macaques; however, our prior studies in Balb/C mice reported CD8^+^ T cell responses in animals immunized with gC2 and gD2 [16]. Phase I/II clinical trials will be required to assess whether CD8^+^ T cell responses will occur in humans.

HSV-2 gD2 is a potent antigen for inducing neutralizing antibodies [39]. In guinea pigs, we noted that the trivalent group had lower neutralizing antibody titers than the gD2 group. Similar gD2 concentrations of 10μg were used in the gD2 alone and the trivalent vaccine preparations. In rhesus macaques, gC2 immunization induced neutralizing antibodies and we reported a similar response to gC2 immunization in guinea pigs [16]; therefore, we expected comparable or higher neutralizing titers in the trivalent group compared with gD2 alone. The most likely explanation for the lower titers in the trivalent group is that we have not yet determined the optimum concentrations of antigens and adjuvants in the vaccine. It appears that the concentrations used in the trivalent vaccine blunted the neutralizing antibody response by diminishing the immunogenicity of gD2. To correct for this shortfall, we immunized some guinea pigs three times with the trivalent vaccine and then gave a booster immunization with gD2 alone (trivalent + gD2 group), which induced neutralizing antibody titers significantly higher than the gD2 alone or trivalent groups. For the trivalent vaccine, the mean endpoint neutralization titer using plasma from rhesus macaques or serum from guinea pigs was approximately 1:640, while the gD2 vaccine induced serum neutralizing antibody titers of 1:1280 in guinea pigs. These high titers compare favorably with the neutralizing antibody titers of 1:160 detected in HSV-2 infected humans and are much higher than the neutralizing antibody titers of 1:29 induced by the GSK gD2 vaccine in humans when sera were tested by the Herpevac Trial investigators and independently in our laboratory [15, 28].

Complement failed to enhance the neutralization of the trivalent group to a greater extent than noted for the gD2 group. This observation differs from our prior results in guinea pigs immunized with gD2 alone compared with gC2 and gD2 in which complement significantly augmented the neutralization of the gC2/gD2 group compared with gD2 alone[16]. The booster dose of gD2 given to the trivalent + gD2 group improved the neutralizing antibody response with and without complement and restored the observation that gC2 immunization enhances the neutralizing titers in the presence of complement. It is possible that the formulation of trivalent antigens and adjuvants not only interfered partially with gD2 immunogenicity, but also blunted responses mediated by gC2.

The trivalent vaccine group outperformed gD2 alone despite inducing lower neutralizing antibody titers with and without complement, suggesting that neutralizing antibody titers are not sufficient to explain the vaccine protection [40]. It is possible that antibody-dependent cellular cytotoxicity contributed significantly to vaccine protection as reported for HSV-2 ΔgD2, a gD2 null live virus vaccine [41, 42]. Alternatively, blocking cell-to-cell spread mediated by antibodies to gE2, or enhanced antigen presentation facilitated by blocking the interaction of gC2 with C3b may account for the improved protection by the trivalent vaccine [10, 21]. Additional studies will be required to assess the immune correlates of protection mediated by the trivalent vaccine.

Reducing the risk of transmission to partners is an important goal for a genital herpes vaccine. The current standard for evaluating risk of transmission is to assess HSV-2 DNA copy number in genital secretions [43–45]. Studies in humans have demonstrated that most episodes of HSV-2 DNA shedding do not contain replication competent virus as determined by failure to isolate virus in cell culture [44]. Our results in guinea pigs are similar to the human studies [12]. The positive predictive value of HSV-2 DNA shedding for isolating replication competent virus was 4% in the trivalent group and 0% in the trivalent + gD2 group, while the negative predictive value was 100% since replication competent virus was never isolated on days that HSV-2 DNA shedding was undetectable. Although the titer of replication competent virus required for transmission to a partner is unknown, we propose that genital shedding of replication competent virus is a more relevant biomarker for risk of transmission than HSV-2 DNA shedding and should be considered as a possible endpoint for a vaccine trial that aims to prevent transmission. The risk of shedding replication competent virus is extremely low when the HSV-2 DNA copy number is <4 log_10_; therefore, limiting vaginal shedding to <4 log_10_ may represent a useful target for future vaccine trials.

An unexpected result was that the mock-infected animals did not shed replication competent virus despite shedding HSV-2 DNA on twice as many days as animals in the gD2 or trivalent groups. A plausible explanation for this observation is that 18/24 (67%) mock-immunized animals died during the acute infection and only eight animals with less severe infection survived. Therefore, the absence of shedding of replication competent virus in mock-immunized animals may represent a selection bias based on the animals available for analysis. In support of this possibility, in another experiment in our laboratory, naïve animals that survived severe intravaginal infection shed HSV-2 DNA on 26/252 (10.3%) days, which is comparable to the mock group in this study; however, replication competent virus was isolated on 5/42 (11.9%) days in contrast to 0/16 days from the mildly ill animals in the current report. Additional studies will be required to further assess the shedding of replication competent virus in the control group and to evaluate in control and immunized animals whether the absence of replication competent virus is because the DNA is naked, fragmented, contains nonviable mutations, or is within an intact virus that is coated by antibodies.

In summary, the trivalent vaccine met our immunogenicity goals in macaques and was highly efficacious in preventing genital lesions in guinea pigs. Administering a booster dose of gD2 minimally enhanced efficacy. Replication competent virus, as a marker for risk of transmission, was isolated from the trivalent vaccine group on 4% of days when trivalent-immunized animals were shedding HSV-2 DNA, which occurred only on 5% of the days, resulting in an overall detection rate of replication competent virus of 0.2%. We propose that the trivalent vaccine is an outstanding candidate for prevention trials in humans.

## Materials and Methods

### Rhesus Studies Ethics Statement

All animals were housed at the Tulane National Primate Research Center in accordance with the Association for Assessment and Accreditation of Laboratory Animal Care International Standards. The Institutional Animal Care and Use Committee (IACUC) of Tulane University approved the use of adult female Chinese rhesus macaques (*Macaca mulatta*), the housing at the Tulane National Primate Research Center, and all macaque procedures described (protocol number P0210). This study was also carried out in strict accordance with the recommendations in the Guide for the Care and Use of Laboratory Animals of the National Institutes of Health (NIH) and with the recommendations of the Weatherall report: “The use of non-human primates in research”. All procedures were performed under anesthesia using ketamine, and all efforts were made to minimize stress, improve housing conditions, and animals were provided with enrichment opportunities including objects to manipulate in the cages, varied food supplements, foraging and task-oriented feeding methods, and interaction with caregivers and research staff. Where indicated, animals were humanely euthanized by ketamine sedation followed by intravenous barbiturate overdose, which is consistent with the recommendation of the Panel on Euthanasia of the American Veterinary Medical Association. The animals were screened for Herpes B virus infection and only Herpes B virus negative animals were used [46].

### Guinea Pigs Ethics Statement

Female Hartley strain guinea pigs (Charles River Laboratories) weighing 275–350 g were housed at the University of Pennsylvania. The Institutional Animal Care and Use Committee of the University of Pennsylvania approved the protocol for these studies (protocol 805187). The protocol adhered to the recommendations in the Institute for Laboratory Animals Research’s “Guide for the Care and Use of Laboratory Animals”. Guinea pigs showing signs of dehydration (>10% weight loss) were treated with subcutaneous saline. In experiment two, animals that developed urinary retention, hind limb weakness or more than two genital lesions during the acute infection (days 1–14) were treated with acyclovir intraperitoneally 50mg/kg once daily to help them recover from the acute infection. Only the mock-immunized animals required acyclovir treatment. In both experiments, meloxicam was used for pain control at the first appearance of genital lesions, and animals were euthanized using Euthasol according to the recommendations of the Panel on Euthanasia of the American Veterinary Medical Association.

### Human Studies Ethics Statement

The Institutional Review Board of the University of Pennsylvania approved the use of the human sera for these studies (protocol 053000).

### Rhesus Macaque Immunizations and Sample Collection

The subunit antigens bac-gC2(426t), bac-gD2(306t) and bac-gE2(24-405t) were prepared in baculovirus as previously described [10]. Originally, four animals were available. Two animals were mock immunized with the CpG oligonucleotide (sequence 5'-TCG TCG TTT CGT TTT GTC GTT-3') (Trilink Inc.) using 500μg CpG and 300μg alum (Alhydrogel, Accurate Chemical and Scientific Corp.), and two animals were immunized with the trivalent vaccine containing 20μg of each antigen (gC2, gD2 and gE2) mixed with 500μg CpG/300μg alum in a total volume of 500μl per immunization given three times at 4-week intervals [47]. The individual antigens for the trivalent vaccine were mixed with CpG and alum and combined immediately prior to immunization. The trivalent group received a booster immunization 28 weeks after the third immunization; while the mock-immunized animals were reassigned to receive 20μg gC2 mixed with 500μg CpG/300μg alum that was given 3 times at one-month intervals. Anticoagulated blood was collected prior to each immunization, prior to intravaginal HSV-2 challenge and terminally. The plasma was stored at -80°C and used for antibody assays while PBMCs were stored in liquid nitrogen and used for T cell assays.

### Rhesus Macaque Challenge Studies

Two naïve animals were added as controls for the trivalent-immunized animals. All four animals were administered 30mg of Depo-Provera intramuscularly and four weeks later they were challenged intravaginally with 1x10^8^ PFU of HSV-2 strain G in 1 ml of DMEM. Seven months after the first challenge, two additional naïve animals were added to the study and all six animals were given Depo-Provera and challenged three weeks later. Animals were challenged again after two weeks and euthanized two weeks after the last challenge.

### Rhesus Macaque Vaginal Fluid Collection

Vaginal secretions were obtained using Weck-Cel sponges that were inserted into the vaginal cavity for 5 minutes and then placed in 300μl of sterile PBS. The sponges were drained of fluid by spinning at 1500 x g for 10 minutes, and stored at −80°C.

### Rhesus Macaque ELISA

ELISA was performed to measure antibody responses in plasma and vaginal secretions [16]. ELISA plates were coated with 100ng of gC2, gD2 or gE2 antigen for plasma ELISA and mucosa IgG antibody studies. For mucosa IgA antibody studies, 33ng of each antigen were combined to coat the wells. The secondary antibodies were HRP-conjugated anti-rhesus IgG or IgA. The endpoint titer was considered the highest dilution of serum resulting in an optical density (OD) of 0.1 and at least 2-fold higher than the OD of samples from mock-immunized animals at that dilution. The initial dilution tested was 1:500. Titers that were negative at 1:500 were plotted as 1:250.

### Rhesus Macaque and Human Neutralizing Antibody Titers

Serial dilutions of plasma from rhesus macaques or sera from humans starting at 1:20 were incubated with 100 PFU of HSV-2 strain MS at 37°C for 1 h, or for some experiments with HSV-1 strain NS. Virus titers were determined by plaque assay on Vero cells. The endpoint neutralization titer was considered the serum dilution that reduced the number of plaques by 50% compared with PBS controls [16]. Mucosa neutralizing antibody titers were performed starting at a 1:10 dilution of vaginal secretions. To determine whether the trivalent vaccine induced neutralizing antibodies to African HSV-2 isolates, 10^5^ to 10^6^ PFU of virus was incubated with a 1:40 dilution of plasma from mock or trivalent immunized macaques for 1 h at 37°C and the virus titer determined by plaque assay on Vero cells (ATCC) [28].

### Rhesus Macaque Flow Cytometry

PBMCs were prepared from immunized animals, cryopreserved and later thawed. The cells were stimulated overnight in complete RPMI-1640 medium containing 10% fetal calf serum (R10) at a concentration of 1x10^6^ cells/ml. *In vitro* stimulation included purified gC2, gD2 and gE2 subunit proteins, R10 media (as a negative control) or Staphylococcal Enterotoxin B (Toxin Technology, Inc., as a positive control) in the presence of 0.5μg/ml of anti-CD28 (28.2, BD Biosciences) and anti-CD49d (9F10, BD Biosciences) monoclonal antibodies [48–50]. Brefeldin A (10 μg/ml, Sigma) was added to cultures one h after stimulation began. After overnight stimulation, live/dead stain was added followed by cell surface markers that were incubated for 30 min. at room temperature. The surface markers used were directly conjugated monoclonal antibodies, CD3 Pacific Blue (SP34-2, BD Biosciences), CD4 APC-H7 (L200, BD Biosciences) and CD8 PE-TexasRed (3B5, Invitrogen). Cells were washed with Dulbecco’s PBS/1% BSA, fixed and permeabilized using Cytofix/Cytoperm (BD Biosciences), washed in Perm Buffer (BD Biosciences), and stained with intracellular monoclonal antibodies, IFNγ PE-Cy7 (4S.B3, BD Biosciences), TNFα Alexa700 (MAb11, BD Biosciences), and IL2 PerCP-Cy5.5 (MQ1-17H12, Biolegend) for 30 min. at room temperature. Cells were washed with PBS and fixed with BD stabilizing fixative buffer (BD Biosciences). Flow cytometry was completed within 24 h using a BD Fortessa instrument (BD Immunocytometry System) and FACSDiva software (BD Immunocytometry System). Data analysis was performed using FlowJo software (version 9.9). Cells were gated first on singlets, followed by lymphocytes, live cells, CD3^+^ T cells and then on CD3^+^CD4^+^ and CD3^+^CD8^+^ T cell subsets. Gated CD4^+^ and CD8^+^ T cells were further analyzed for cytokine production. Cytokine responses were considered positive if the response was two-fold higher than the medium control and > 0.05%.

### Rhesus Macaque Histopathology and Immunohistochemistry

2x3 mm punch biopsies were obtained at strategic time points after vaginal HSV-2 challenge and fixed in neutral buffered formalin and embedded in paraffin for histopathology. Sections were stained with H&E and evaluated by a pathologist for inflammation and lesions. For immunohistochemistry, formalin-fixed, paraffin embedded sections were prepared and stained as previously described with slight modification [51]. Briefly, sections were de-paraffinized and antigens “unmasked” using high temperature antigen retrieval in a steam chamber (Black and Decker Flavor Center Steamer Plus) with 0.01 M citrate buffer and Dako Target Retrieval Solution. Slides were cooled, washed three times in PBS, and incubated with Dako Dual Endogenous Enzyme Block solution followed by Protein Blocking Solution (Dako Inc.). Dako rabbit anti-HSV polyclonal antibody was used as the primary antibody followed by EnVision Dual Link System HRP secondary antibody for 45 minutes and developed using diaminobenzidine (DAB) as the chromagen. For positive controls, sections of skin or tongue from macaques infected with simian herpes B virus were used to assess specificity. For negative controls, slides from uninfected macaque tissues processed as above were included in each run. In addition, sections incubated with and without primary or secondary antibodies were included as negative controls. No non-specific background staining was detected in any controls. Slides were mounted with fluorescent mounting medium (Dako Inc.) and imaged using Inform software.

### Rhesus Macaque C3b and IgG Fc Blocking Assays

ELISA was used to evaluate antibody responses to gC2 and gE2 that block the interaction of gC2 with C3b and gE2 with IgG Fc [10, 16]. For C3b blocking assays, wells of a 96-well High Binding Costar microtiter plate (Corning Incorporated) were coated with purified human C3b at 50ng/well. For IgG Fc blocking assays, non-immune human IgG purified from an HSV-1/HSV-2 seronegative donor, was used to coat wells at 1μg/well in Na bicarbonate binding buffer (pH 8.5). The C3b or IgG was incubated for 1 h at room temperature and then blocked for 2 h at room temperature with 5% nonfat milk in PBS 0.05% Tween-20. Purified IgG was incubated with 50ng gC2 or 400ng gE2 for 1 h at 37°C, and added to C3b or IgG coated wells, respectively for 1 h. Bound gC2 was detected with a polyclonal rabbit anti-gC2 serum, R80 followed by HRP-conjugated goat anti-rabbit IgG, while bound gE2 was detected using a polyclonal rabbit anti-gE2 serum, R265 followed by HRP-conjugated donkey anti-rabbit IgG.

### Antigens, Immunizations and Challenge of Guinea Pigs

Guinea pigs were bled from a hind limb saphenous vein. Immunizations were performed intramuscularly in the right hind calf muscle three times at 2-week intervals. Animals were mock-immunized with the CpG oligonucleotide (5’-TCGTCGTTGTCGTTTTGTCGTT-3’) (Trilink Inc.) using 100μg CpG/guinea pig and 150μg alum (Alhydrogel, Accurate Chemical and Scientific Corp.), or immunized with 10μg gD2 mixed with 100μg CpG/guinea pig and 150μg alum, or immunized with the trivalent vaccine containing 10μg each of gC2, gD2 and gE2 mixed with100μg CpG/guinea pig and 450μg alum in a total volume of 50μl per immunization [16]. The individual antigens for the trivalent vaccine were mixed with CpG and alum and combined immediately prior to immunization.

The results are combined observations from two separate experiments for mock, gD2 and trivalent immunized animals. A fourth immunization with gE2 alone was given to the trivalent group in experiment one due to lower than expected antibody responses to gE2. A new preparation of subunit gE2 antigen was used for the fourth immunization and for the three immunizations in experiment two. No differences in outcome were observed between the trivalent group that received the extra dose of gE2 in experiment one and three immunizations in experiment two. Based on lower than expected neutralizing antibody titers in experiment one for the trivalent group, we added an additional vaccine group that received three immunizations of the trivalent vaccine followed by a gD2 booster dose two weeks later containing 10μg gD2, 100μg CpG and 150μg alum (trivalent + gD2 group, n = 9).

Animals were challenge intravaginally with 5x10^5^ PFU of HSV-2 strain MS two weeks after the final immunization in experiment one or four weeks after the final immunization for mock-, gD2- and trivalent-immunized animals in experiment two, and two weeks after the final immunization for the trivalent + gD2 group. Animals were scored for acute disease on days 1 to 14 and recurrent genital lesions on days 15 to 60. Scores of 0 to 4 were as follows: 0 reflects no lesions, 1 represents one isolated lesion, 2 reflects two or more separate lesions, 3 represents coalesced lesions, and 4 reflects ulcerated lesions. Urinary retention and hind leg weakness were recorded. Two investigators evaluated the guinea pigs for disease and the lesion scores that were assigned were based on consensus. The investigators were blinded as to vaccination status for the second experiment but not for the first experiment. The results of the two experiments were comparable in that the trivalent vaccine outperformed gD2 and both vaccines outperformed mock in each experiment. Vaginal swabs were performed daily on days 29–49 post-challenge to monitor for HSV-2 DNA copy number and replication competent virus. The swabs were stored at -80°C prior to culture or processing for HSV-2 DNA by qPCR.

### Guinea Pig ELISA and Neutralizing Antibodies

ELISA plates were coated with 50ng of gC2, gD2 or gE2 subunit antigen, incubated with guinea pig serum at a 1:1000 dilution, followed by HRP-conjugated anti-guinea pig IgG [29]. Neutralizing antibody titers were determined as described above, except that serum was used instead of plasma and 10% human serum from an HSV-1/HSV-2 seronegative donor was added as a source of complement [16].

### Virus Cultures from Guinea Pig Vaginal Swabs

On days 29–49 post-challenge, vaginal swabs were collected in one ml Dulbecco’s modified Eagle’s medium (DMEM) containing 5% fetal bovine serum supplemented with 25μg/ml of vancomycin [16]. 150μl of media from the swab was added to a Vero cell monolayer in a 24-well plate for one hour at 37°C. Cells were overlaid with 0.5% methylcellulose in complete DMEM supplemented with vancomycin. Plaques were counted 72 hours later. The lower limit of detection was 6.7 PFU/ml.

### Real-time qPCR on Guinea Pig Vaginal Swabs

DNA was isolated from 200μl of guinea pig vaginal swab material using DNeasy blood and tissue kits (Qiagen) [16]. HSV-2 DNA copy number was determined using purified HSV-2 DNA (Advanced Biotechnologies) and based on a standard curve that was generated with 50,000, 5,000, 500, 50, and 5 copies of DNA and run in triplicate. Each guinea pig sample was analyzed in duplicate. Samples with <150 copies/ml by 40 cycles or only positive in one of two wells were reported as negative. Primer and probe sequences for HSV-2 Us9 were: primer forward, 5′-GGCAGAAGCCTACTACTCGGAAA-3′, and reverse 5′-CCATGCGCACGAGGAAGT-3′, and probe with reporter dye 5′-FAM-CGAGGCCGCCAAC-MGBNFQ-3′ (FAM, 6-carboxyfluorescein). All reactions were performed using TaqMan gene expression master mix (Applied Biosystems) and data was collected and analyzed on an ABI 7500 Fast machine.
